# Great American Smokeout — November 16, 2017

**DOI:** 10.15585/mmwr.mm6644a1

**Published:** 2017-11-10

The American Cancer Society’s Great American Smokeout is an annual event that encourages smokers to make a plan to quit smoking (*1*). The 42nd annual Great American Smokeout will be held on November 16, 2017.

In the more than 50 years since the Surgeon General’s first report on smoking and health, cigarette smoking among U.S. adults has been reduced by approximately half. Nonetheless, since 1964, the year of that first report, an estimated 20 million persons have died because of smoking. Smoking remains the leading preventable cause of disease, disability, and death in the United States (*2*).

About two out of three adult smokers want to quit smoking cigarettes, and approximately half of smokers made a quit attempt in the preceding year (*2*). However, in 2016, more than one in seven U.S. adults were current cigarette smokers (*3*). Getting effective help through counseling and use of medications can increase the chances of quitting by as much as threefold (*4*).

Information and support for quitting smoking is available by telephone at 800-QUIT-NOW (800–784–8669). CDC’s Tips From Former Smokers campaign offers additional quit resources at https://www.cdc.gov/tips.
